# ﻿Lycoris × xiui, a new wild nothospecies from Anhui, China, a hybrid between *L. longituba* and *L. sprengeri* (Amaryllidaceae)

**DOI:** 10.3897/phytokeys.269.175227

**Published:** 2026-01-16

**Authors:** Ying-Feng Hu, Zhen-Zhen Xu, Ke-Run Zhu, Xiao-Yu Si, Jian-Wen Shao, Si-Yu Zhang, Ju Tang

**Affiliations:** 1 College of Life Sciences, Anhui Normal University, Wuhu 241000, China; 2 Environmental Technology Limited Company, Huashe Design Group, Nanjing 210014, China; 3 The Anhui Provincial Key Laboratory of Biodiversity Conservation and Ecological Security in the Yangtze River Basin, Anhui Normal University, Wuhu 241002, China; 4 College of Civil and Architecture Engineering, Chuzhou University, Chuzhou 239000, China

**Keywords:** chloroplast genomes, interspecific hybridization, karyotype, plant systematics

## Abstract

A new wild nothospecies, Lycoris × xiui S.Y.Zhang, Ying F.Hu & J.W.Shao, from Anhui Province in eastern China, is described and illustrated based on complete chloroplast genome, morphological, and karyotype evidence. This taxon resulted from the hybridization of *L. longituba* and *L. sprengeri* and is currently known only from Langya Mountain. Phylogenetic analysis of the chloroplast genome revealed that *L. sprengeri* served as the maternal parent of all hybrid individuals sequenced. It is characterized by a karyotype of 2*n* = 19 = 3V + 16I, a perigone tube typically 1.5–3.5 cm long, and flowers usually pink to pale pink (rarely white or pale yellow), with tepal apices sometimes pale blue. These characteristics differ markedly from those of both putative parents; therefore, this study formally names the taxon and provides a detailed morphological description and diagnosis.

## ﻿Introduction

The emergence of natural hybrids reflects the existence of incomplete reproductive isolation between native species in wild environments, representing a frequently occurring phenomenon in nature that is particularly prevalent in the plant kingdom (Stebbins 1971; Arnold 1997; Rieseberg 1997; Mallet 2007; Soltis and Soltis 2009; Wu et al. 2022). Stable hybrid taxa hold significant taxonomic importance and, in accordance with the International Code of Nomenclature for algae, fungi, and plants, warrant recognition at the nothospecies rank (ICN; Turland et al. 2025). The genus *Lycoris* in the Amaryllidaceae family exemplifies a group characterized by widespread natural hybridization (Hsu et al. 1994; Shi et al. 2006). This genus, primarily distributed across Pan-East Asia, currently comprises 35 accepted species. However, only 11 species are considered diploid entities with morphologically stable wild populations capable of natural regeneration through sexual reproduction (Hsu et al. 1994; Ji and Meerow 2000; Lu et al. 2020; Lou et al. 2022; Zhang et al. 2022a, 2022b). Of the remaining taxa, six species have been identified as nothospecies with clearly defined parental species, such as *L. × hubeiensis* Kun Liu and L. × jinzheniae S.Y.Zhang, P.C.Zhang & J.W.Shao (Hsu et al. 1994; Meng et al. 2018; Zhang et al. 2022a; Quan et al. 2024). For most remaining nothospecies, the identity of their parental species remains the subject of future research. Although their hybrid origins were often overlooked when these names were originally established, in accordance with the ICN, most of these names may arguably retain their status as species-level taxa (Turland et al. 2025).

**Figure 1.**
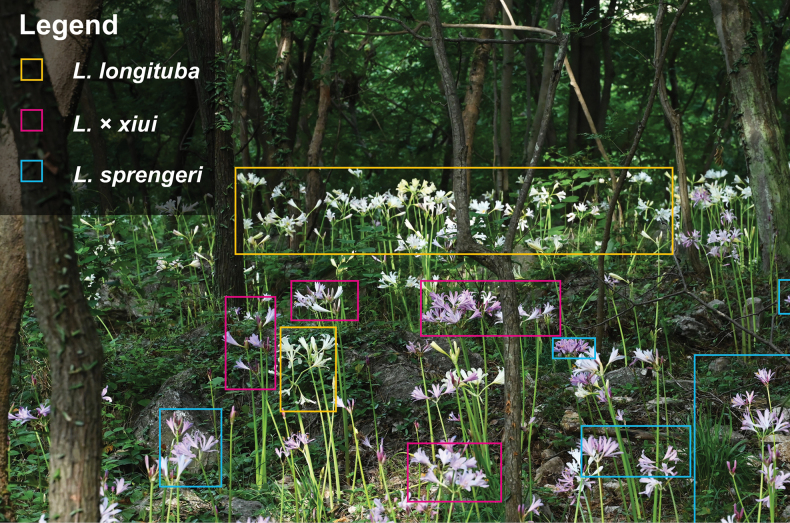
Wild mixed population of *Lycoris longituba*, *L. sprengeri*, and L. × xiui.

**Figure 2.**
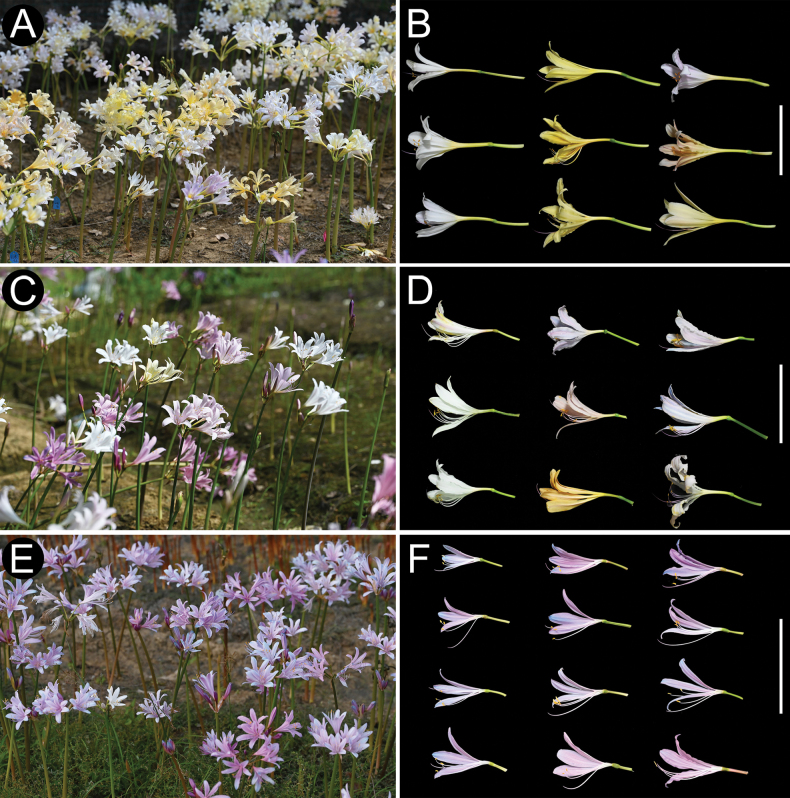
Morphological characteristics of *Lycoris longituba*, *L. sprengeri*, and L. × xiui under homogeneous garden cultivation conditions. **A, B.***L. longituba*; **C, D.**L. × xiui; **E, F.***L. sprengeri*. Left column: population photographs; right column: close-ups of flower variation. Scale bar length = 10 cm.

In July 2022, during fieldwork focused on *Lycoris* specimen collection, our research group unexpectedly discovered a distinctive plant community on Langya Mountain in Chuzhou City, Anhui Province, China. This population included *L. sprengeri* Comes ex Baker, *L. longituba* Y.Hsu & G.J.Fan, and an unidentified *Lycoris* taxon exhibiting morphology intermediate between these two species—unlike any known species (Figs 1, 2). Critically, our prior seven-year investigations had never documented the sympatric occurrence of *L. longituba* and *L. sprengeri*. We therefore hypothesized that the unknown taxon (putative new nothospecies) represented a novel natural hybrid between *L. longituba* and *L. sprengeri*. To test this hypothesis, we conducted a comprehensive study integrating morphological observations, chromosomal examination, and phylogenomic reconstruction based on complete chloroplast genomes. This work aims to clarify the hybrid origin and taxonomic status of this newly discovered entity, thereby contributing to the understanding of natural hybridization in *Lycoris*.

## ﻿Materials and methods

### ﻿Plant collection and morphological analysis

From July to September 2022, inflorescences and bulbs of the putative new nothospecies, *Lycoris longituba*, and *L. sprengeri* (50 individuals per species) were collected from Langya Mountain. One fully bloomed flower per inflorescence was randomly selected on-site and measured for perigone tube length, tepal length, tepal width, style length, and filament length using a digital vernier caliper (accuracy: 0.01 mm). Bulbs were cultivated in the Homogeneous Garden, Fengyang County, Chuzhou City, for long-term observation of morphological stability, seed-setting status, and chromosome studies. Morphological data from the three species were processed using Origin 2021 software. Principal component analysis (PCA) was performed, and results were visualized as box plots overlaid with normal distribution curves. Statistical significance of differences was analyzed in SPSS ver. 25 using letter labeling to denote homogeneous subgroups (Zhang et al. 2022a).

### ﻿Karyotype and pollen viability observation

In June 2023, 3–5 bulbs per species were buried in moist sand to induce root growth. Newly sprouted root tips (≈1 cm) were excised for chromosome observation. After thorough washing, root tips were soaked in 0.2% colchicine solution at room temperature for 12 h, rinsed, and fixed in Carnoy’s fluid (anhydrous ethanol: glacial acetic acid = 3:1) at 4 °C for 12 h. Fixed tissues were rinsed, hydrolyzed in 1 mol/L HCl at 60 °C for 15 min, washed, stained with modified carbol fuchsin for 30 min, and squashed for microscopic examination. Chromosomal karyotypes were determined by analyzing 30 well-spread metaphase cells per species (Zhang et al. 2022a, 2022b). With reference to the method of Traub and Moldenke (1949), chromosomes were classified using “V” to represent metacentric or submetacentric chromosomes and “I” to represent acrocentric chromosomes.

Observation of pollen viability was conducted in July 2023. At 8:00 AM, pollen was collected from freshly dehisced anthers, placed on a glass slide, mixed with a 0.5% TTC solution, and kept at room temperature for 15 min. For each sample, five fields of view were observed to examine staining status (Oberle and Watson 1953; Zhang et al. 2021).

### ﻿Acquisition, annotation, comparison, and phylogenetic analysis of chloroplast genomes

Total genomic DNA was extracted from silica gel–desiccated leaf material using a modified CTAB protocol (Doyle and Doyle 1987; Larridon et al. 2015). DNA quality after PCR amplification was assessed using a NanoDrop 1000 spectrophotometer (Thermo Fisher Scientific), complemented by 1% agarose gel electrophoresis. Library preparation and genome skimming were conducted at the Germplasm Bank of Wild Species (Kunming, China) on an Illumina HiSeq 6000 platform. Approximately 3 Gb of raw sequencing data per sample underwent chloroplast genome assembly using GetOrganelle v1.7.1 (Jin et al. 2020), with subsequent annotation performed using PGA (Qu et al. 2019).

**Figure 3.**
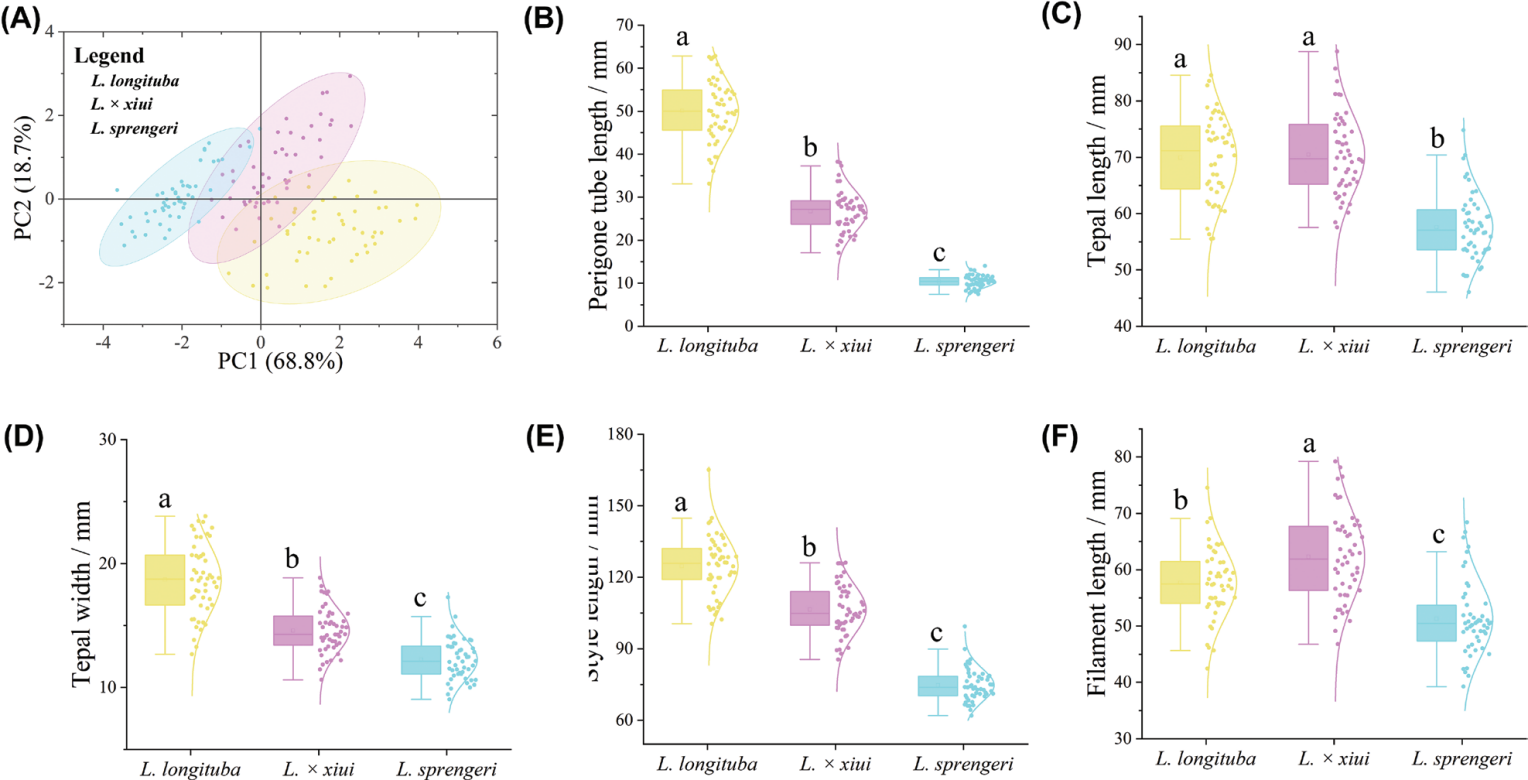
Morphological statistical analysis of *Lycoris longituba*, *L. sprengeri*, and L. × xiui. **A.** Principal component analysis based on five floral morphological indices; circles represent 95% confidence intervals; **B–F.** Descriptive statistical analysis based on five floral morphological indices: **B.** Perigone tube length; **C.** Tepal length; **D.** Tepal width; **E.** Style length; **F.** Filament length. In the boxplot, the horizontal line indicates the median, and the lower and upper edges of the box represent the first and third quartiles, respectively. Boxplots marked with different letters indicate statistically significant differences (post hoc test, P < 0.05).

The final dataset comprised four novel chloroplast genomes of Lycoris × xiui generated in this study (Table 1) and 16 published complete chloroplast genomes of fertile diploid congeners retrieved from NCBI (accession details provided in Table 1, Fig. 3), with *Narcissus poeticus* L. (accession: MH706763) designated as the outgroup (Könyves et al. 2018). Nucleotide sequences were aligned using MACSE v2 (Ranwez et al. 2018). Phylogenetic reconstruction was implemented using both maximum likelihood (ML) and Bayesian inference (BI) frameworks. The optimal nucleotide substitution model, identified using ModelFinder (Kalyaanamoorthy et al. 2017), informed subsequent analyses. ML trees were inferred with IQ-TREE v1.6.8 (Nguyen et al. 2015) under the GTR+G+I model, with nodal support evaluated via 1,000 bootstrap replicates. Bayesian analysis conducted in MrBayes v3.2.6 (Ronquist et al. 2012) employed eight Markov chains running for 20,000,000 generations, with sampling every 1,000 generations. All analytical workflows were managed within PhyloSuite (Zhang et al. 2020).

**Table 1.** Basic characteristics and GenBank accession numbers of chloroplast genomes used in this study.

Species name	GenBank number	Length (bp)
Lycoris × xiui	PV839564	158,513
Lycoris × xiui	PV839565	158,513
Lycoris × xiui	PV839566	158,513
Lycoris × xiui	PV839567	158,513
*Lycoris aurea*	MN831471	158,690
*Lycoris chinensis*	OP034613	158,354
*Lycoris insularis*	OP034614	158,366
*Lycoris insularis*	OP034615	158,360
*Lycoris longifolia*	ON960856	158,413
*Lycoris longituba*	MK353218	158,633
*Lycoris longituba*	MN841763	159,728
*Lycoris radiata*	MT348454	158,355
*Lycoris radiata*	MN158120	158,335
*Lycoris sanguinea*	MK353220	158,761
Lycoris sanguinea var. kiushiana	ON611637	158,714
Lycoris sanguinea var. koreana	ON611638	158,727
*Lycoris sprengeri*	OP034616	158,359
*Lycoris sprengeri*	OP034617	158,366
*Lycoris sprengeri*	OP034618	158,366
*Lycoris sprengeri*	OP034619	158,151
*Narcissus poeticus*	MH706763	160,099

## ﻿Results

### ﻿Morphological and fertility comparison

Morphological observations based on two years of common-garden cultivation indicated that flowering forms and colors remained stable annually, with no variation among individuals of the putative new nothospecies, *Lycoris longituba*, and *L. sprengeri*. Measurements and observations of twelve bulb, leaf, and floral characteristics, with reference to previous studies, revealed that the putative new nothospecies differs morphologically from its parents in numerous traits; specific morphological differences are presented in Table 2. Principal component analysis (PCA) of five floral characteristics (Fig. 3A) showed that the first two principal components captured a high cumulative variance (PC1 + PC2 = 87.5%), encompassing most of the original data information and therefore suitable for analyzing differences among samples. In the plot, the 95% confidence ellipses for *L. longituba* and *L. sprengeri* did not overlap, whereas the confidence ellipse for the putative new nothospecies overlapped with those of both parents, with some individuals falling within the confidence regions of the other two taxa. Descriptive statistical analysis demonstrated significant differences among the three taxa for perigone tube length, tepal width, style length, and filament length (Fig. 3C–F). For perigone tube length, tepal width, and style length, the mean values of the putative new nothospecies were intermediate between those of the other two taxa. Notably, for filament length, the mean value of the putative new nothospecies was significantly higher than those of both parents.

**Table 2.** Comparison of Lycoris × xiui, *L. longituba*, and *L. sprengeri*.

Characters	L. × xiui	*L. longituba*	*L. sprengeri*
Bulb diameter	3.5–5 cm	4–6 cm	3–4 cm
Leaf length	30–45 cm	35–45 cm	30–40 cm
Leaf width	1.5–2.5 cm	2–4 cm	1–1.4 cm
Leaf color	green to dark green	dark green	pale green
Degree of leaf twist	slight	no	strong
Leaf apex color	red	green	red
Scape height	40–70 cm	60–80 cm	35–55 cm
Flower color	pink to pale pink, occasionally white or pale yellow, with pale blue apex	white, lemon-yellow, or cream-yellow (very rarely pale pink)	deep pink, with blue apex
Perigone tube length	1.5–3.5 cm	3–6.5 cm	0.5–1.3 cm
Tepal length	5.5–9 cm	5.5–9 cm	4–7 cm
Tepal width	1–2 cm	1.3–2.5 cm	0.8–1.7 cm
Style length	8–13 cm	9–15 cm	6–10 cm
Filament length	4.5–8 cm	4–7.5 cm	3.5–7 cm

During common-garden cultivation, observations of seed setting in the three taxa revealed that both *Lycoris longituba* and *L. sprengeri* set seeds normally. Possibly due to favorable cultivation conditions, a high proportion of fruits developed and reached maturity—unlike wild populations, in which a certain proportion of plants exhibit abortive seeds. In contrast, the scapes of the putative new nothospecies withered and aborted within two weeks after flowering. We further conducted simple artificial pollination experiments, including self-pollination within the putative new nothospecies and cross-pollination by applying pollen from *L. longituba* and *L. sprengeri* to the putative new nothospecies, as well as pollen from the putative new nothospecies to *L. longituba* and *L. sprengeri*. Each combination involved 2–5 plants, but all combinations resulted in abortion. Therefore, we conclude that the putative new nothospecies is incapable of producing viable seeds.

### ﻿Karyotype and pollen viability

**Figure 4.**
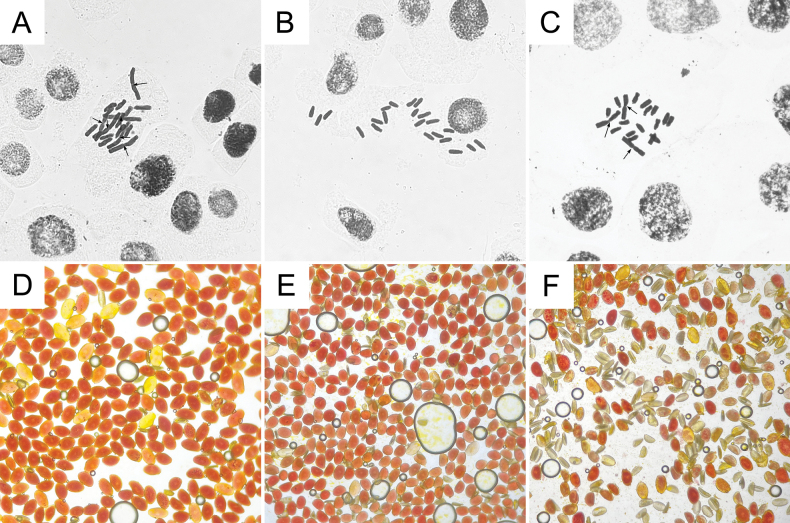
Chromosomes and pollen viability of *Lycoris longituba*, *L. sprengeri*, and L. × xiui. **A.** Chromosomes of *L. longituba*; **B.** Chromosomes of *L. sprengeri*; **C.** Chromosomes of L. × xiui; **D.** Pollen viability of *L. longituba*; **E.** Pollen viability of *L. sprengeri*; **F.** Pollen viability of L. × xiui. Arrows in chromosome images indicate the centromere positions of “V”-shaped chromosomes.

The results indicate that the karyotype formula of *Lycoris longituba* is 2*n* = 16 = 6V + 10I, indicating a haploid chromosome set of *n* = 8 = 3V + 5I. In contrast, *L. sprengeri* has a karyotype of 2*n* = 22 = 22I, with a haploid set of *n* = 11 = 11I. The karyotype of the putative new nothospecies is exactly equal to the sum of one haploid set from each of the two parental species, i.e., 2*n* = 19 = 3V + 16I (Fig. 4A–C).

Observation of pollen viability shows that the pollen of *Lycoris longituba* and *L. sprengeri* is mostly plump and stains orange-red, whereas a large proportion of the pollen from the putative new nothospecies is shrunken, with only a fraction staining successfully (Fig. 4D–E).

### ﻿Molecular phylogenetic relationship

**Figure 5.**
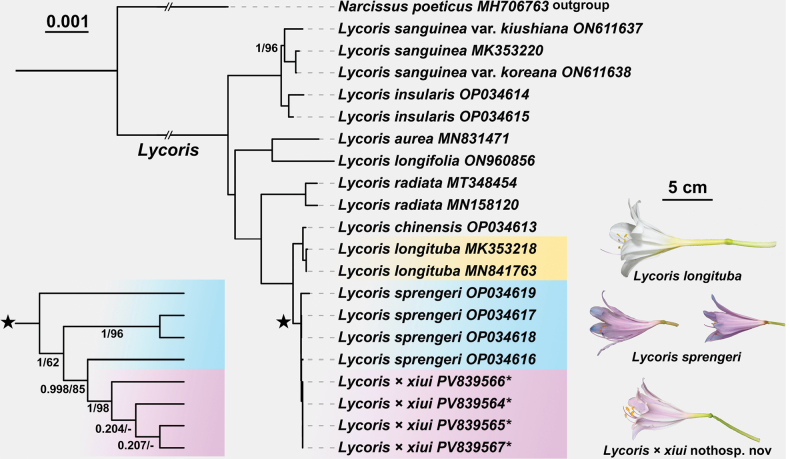
Bayesian phylogenetic tree of *Lycoris* species based on chloroplast genomes from 20 individuals. Numbers above branches represent Bayesian posterior probability (BPP)/maximum likelihood bootstrap (BS) values; branches without labeled support values indicate BPP = 1.0 and BS = 100.

The Bayesian phylogenetic tree constructed based on chloroplast genome data (Fig. 5) was highly consistent in topology with the ML tree, differing only among the three terminal individuals of the putative new nothospecies; therefore, BS values were not labeled for these branches. Overall, most nodes in the inferred tree exhibited high support values (BPP = 1.0, BS = 100), except for several nodes within clades marked with “black star,” which showed relatively low support. In the tree, *L. longituba* and *L. chinensis* formed a clade. Notably, all four individuals of the putative new nothospecies were completely nested within the clade comprising *L. sprengeri* individuals from different localities, together forming a highly supported monophyletic group. However, the key branches supporting the clustering of the putative new nothospecies with *L. sprengeri* generally showed low support values, indicating limited sequence divergence among their chloroplast genomes.

## ﻿Discussion

In summary, our findings indicate that the putative new nothospecies has so far been discovered only in the zone of overlap between populations of *L. longituba* and *L. sprengeri*, currently confirmed only at Langya Mountain. Morphologically, multiple characteristics of the putative new nothospecies, as well as its position in PCA clustering, are intermediate between those of *L. longituba* and *L. sprengeri*. Karyotype observations reveal that the karyotype of the putative new nothospecies is precisely equal to the sum of one haploid chromosome set from each of *L. longituba* and *L. sprengeri*. Pollen viability assessment shows that the pollen of *L. longituba* and *L. sprengeri* is predominantly plump and viable, consistent with the characteristics of fertile progenitor species, whereas the pollen of the putative new nothospecies is often shrunken. This pattern is likely attributable to its aneuploid nature, which leads to irregular synapsis during meiosis. Molecular phylogenetic analysis further indicates that the putative new nothospecies shares the same chloroplast genome as *L. sprengeri*. According to the principle of maternal inheritance of chloroplast DNA in angiosperms (Corriveau and Coleman 1988; Mogensen 1996; Zhang et al. 2003), *L. sprengeri* is therefore inferred to be the maternal parent of the putative new nothospecies.

Based on the evidence presented above, we conclude that the data sufficiently demonstrate that the putative new nothospecies is a hybrid derivative of *L. longituba* and *L. sprengeri*. However, owing to nonviable sexual reproduction, it cannot act as a bridge facilitating gene flow between the two parental species. Although the putative new nothospecies is incapable of viable sexual reproduction, it can persist in nature through vegetative propagation via bulb division. In accordance with the principles of documenting this hybridization event and respecting natural patterns, this study formally describes the putative species as Lycoris × xiui S.Y.Zhang, Ying F.Hu & J.W.Shao.

Regarding filament length, which is greater in Lycoris × xiui than in either parent, this pattern may be explained by heterosis occurring during plant hybridization (Shull 1908; Rieseberg et al. 1999; Hochholdinger and Baldauf 2018). Furthermore, the fact that all four randomly sequenced individuals of L. × xiui shared the same maternal parent (*L. sprengeri*) may indicate the presence of asymmetric hybridization in this cross. A similar phenomenon has been reported in hybrids between *L. chinensis* and *L. insularis* (Zhang et al. 2022a, 2022b). Whether asymmetric hybridization exists, or is widespread, within the genus *Lycoris*, and the underlying mechanisms responsible for this pattern, require further investigation.

### ﻿Taxonomic treatment

#### ﻿ Lycoris × xiui

Taxon classificationPlantaeAsparagalesAmaryllidaceae

S.Y.Zhang, Ying F.Hu & J.W.Shao nothosp. nov.

E9CDCF42-D36F-5061-878F-AFE0E023CEB6

urn:lsid:ipni.org:names:77375126-1

Fig. 6

= Lycoris longituba Y. Hsu & G. J. Fan × Lycoris sprengeri Comes ex Baker.

##### Type.

China. • Anhui, Chuzhou City, Nanqiao District, Langya Mountain, 32°16'33.6"N, 118°14'42"E, under the deciduous broad-leaved forest on the hillside, 85 m a.s.l., 24 July 2022, *S.Y. Zhang, ZSY202208001* (holotype: ANUB100125!; isotypes: ANUB100126!, ANUB100127!, CSH0221073!, CSH0221074!, CSH0221075!).

**Figure 6.**
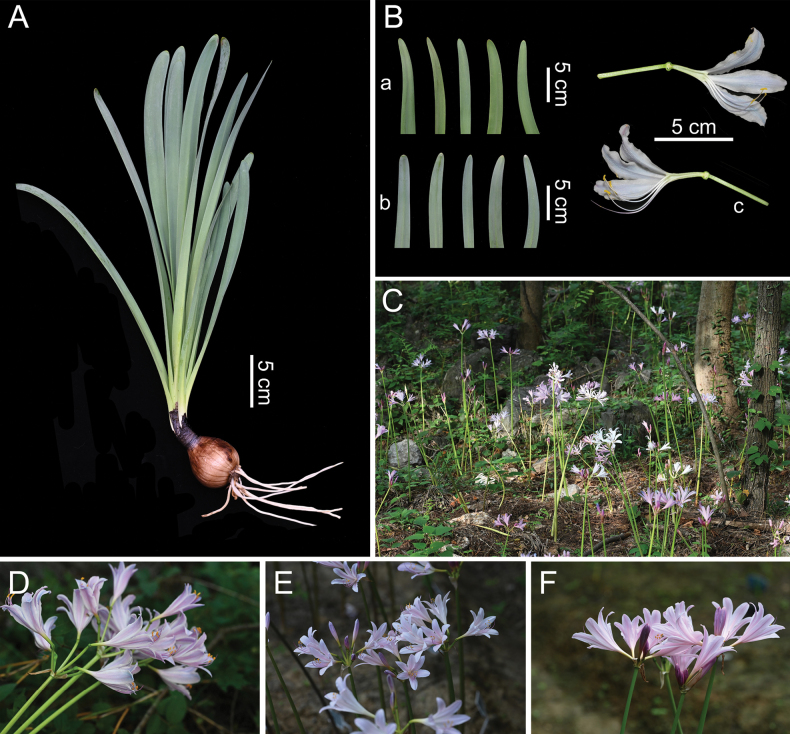
Morphology of Lycoris × xiui. **A.** Vegetative body; **Ba.** Leaf upper surface; **Bb.** Leaf lower surface; **Bc.** Longitudinal section of flower; **C.** Habitat; **D–I.** Floral close-ups of different individuals.

##### Diagnosis.

The new nothospecies resembles its parental taxa (*Lycoris longituba and L. sprengeri*), but is distinguished by its perigone tube typically measuring 1.5–3.5 cm in length and flowers that are usually pink to pale pink, rarely white or pale yellow, with tepal apices sometimes pale blue. In contrast, *L. sprengeri* exhibits a perigone tube less than 1.3 cm long, deep pink flowers, and tepal apices that are usually distinctly blue, while *L. longituba* possesses a notably longer perigone tube (3–6.5 cm), flowers that are white, lemon-yellow, or cream-yellow (very rarely pale pink), and tepal apices that never display a blue coloration.

##### Description.

Perennial herb. Bulb ovoid, 3.5–5 cm in diameter, covered with brown cataphylls bearing fine longitudinal striations. Leaves linear, usually 6–9(–13), apex obtuse, emerging in winter to early spring, 30–45 cm long, 1.5–2.5 cm wide, green to dark green, midrib slightly sunken, surface thinly white-pruinose. Scape 40–70 cm high, green; spathe bracts 2, lanceolate, ca. 4 cm long, 8–15 mm wide. Umbels with 5–7 flowers; pedicels 1.5–3 cm long, ca. 3 mm in diameter; flowers typically pink to pale pink, occasionally white or pale yellow, sometimes with pale blue tepal apices. Tepals oblanceolate, 5.5–9 cm long, 1–2 cm wide, apex slightly recurved and occasionally faintly crisped; perigone tube pale green, ca. 15–35 mm long. Filaments 4.5–8 cm long, white to pale pink, slightly exceeding tepals; anthers yellow, 3–5 mm long; style 8–13 cm long, pale pink with purple apex. Ovary spherical, green, 5 mm in diameter. Capsules trigonous, failing to produce seeds at maturity.

##### Phenology.

Flowering from late July to late August; leaves growing from late February to early March.

##### Distribution and habitat.

Currently confirmed only from the type locality (Chuzhou City, eastern Anhui Province), but potentially occurring in adjacent areas of Nanjing City and Zhenjiang City, Jiangsu Province. It grows in deciduous broad-leaved forests on limestone hills, in mixed populations where *Lycoris sprengeri* (eastern Hubei, central-eastern Anhui, central Jiangsu) and *L. longituba* (central-eastern Anhui, central Jiangsu) co-occur.

##### Etymology.

The specific epithet commemorates the renowned literary figure of China’s Northern Song Dynasty, Xiu Ouyang (欧阳修), whose iconic work “The Old Drunkard’s Arbor” (《醉翁亭记》) has made the discovery site of this nothospecies—Langya Mountain—nationally famous. The Chinese name is designated as 亭城石蒜 (Tíng Chéng Shí Suàn). Tíng Chéng (亭城) is the alias of Chuzhou City, where this new nothospecies was discovered. The historical culture of Chuzhou is closely intertwined with Langya Mountain and the Old Drunkard’s Pavilion (醉翁亭). Shí Suàn (石蒜), the Chinese name of the genus *Lycoris*, literally means “a garlic-like plant growing among rocks.”

##### Reproduction.

This nothospecies is functionally seed-sterile and reproduces exclusively through natural vegetative propagation via bulb division. A single bulb typically forms a cluster of 2–3 daughter bulbs after two growing seasons.

## Supplementary Material

XML Treatment for Lycoris × xiui

## ﻿Additional information

### Use of AI

No use of AI was reported.

### Author ORCIDs

Ying-Feng Hu https://orcid.org/0000-0002-1549-4387

Si-Yu Zhang https://orcid.org/0000-0003-2674-7340

Ju Tang https://orcid.org/0009-0008-8637-0286

### Data availability

All of the data that support the findings of this study are available in the main text.
